# Promoting Effects on Proliferation and Chondrogenic Differentiation of Bone Marrow-Derived Mesenchymal Stem Cells by Four “Kidney-Tonifying” Traditional Chinese Herbs

**DOI:** 10.1155/2015/792161

**Published:** 2015-06-07

**Authors:** Bin Cai, Ai-guo Zhang, Xian Zhang, Wen-jie Ge, Guo-da Dai, Xiang-ling Tan, Gopaul Roodrajeetsing, Jian-ping Cai

**Affiliations:** ^1^Department of Colorectal Surgery, First Affiliated Hospital, Guangxi Medical University, Nanning, Guangxi 530021, China; ^2^Department of Pediatric Surgery, Wuxi People's Hospital, Wuxi, Jiangsu 214000, China; ^3^Department of Orthopedics, Wuxi Hospital of Traditional Chinese Medicine, Wuxi, Jiangsu 214000, China; ^4^Life School, Nantong University, Nantong, Jiangsu 226000, China; ^5^Department of Neurosurgery, First Affiliated Hospital, Guangxi Medical University, Nanningm, Guangxi 530021, China

## Abstract

Traditional Chinese medicine can promote the proliferation of bone marrow-derived mesenchymal stem cells (BMSCs). We chose four “Kidney-tonifying” Chinese herbal medicines, Radix Astragali, Salvia, Herba Epimedii, and Saussurea Involucrata, to evaluate whether they had positive effects on the proliferation of BMSCs and TGF-*β*1-induced chondrogenic differentiation of BMSCs. The four Chinese herbal medicines were intragastrically administered to Sprague-Dawley rats, respectively, to prepare drug-containing serums of corresponding Chinese herbs. BMSCs were isolated, cultured, and exposed to culture solution containing 1%, 5%, 10%, and 15% (v/v) Radix Astragali-, Salvia-, Herba Epimedii-, and Saussurea Involucrata-containing serum, respectively. TGF-*β*1-induced BMSCs were addressed in the same manner. Collagen type II protein was assessed by immunofluorescence methods. To assess whether the drug-containing serums had positive effects on the proliferation of BMSCs and TGF-*β*1-induced BMSCs, MTT method was assessed. The proliferation of BMSCs was significantly enhanced when exposed to culture solutions containing 1% and 5% Radix Astragali-, 1% and 5% Salvia-, 5% Herba Epimedii-, and 1%, 5%, and 10% Saussurea Involucrata-containing serum. The proliferation of TGF-*β*1-induced BMSCs was significantly enhanced when exposed to 1%, 5%, and 15% Radix Astragali-, 10% and 15% Salvia-, 5%, and 15% Herba Epimedii-, and 1%, 5%, and 10% Saussurea Involucrata-containing serum.

## 1. Introduction

Bone marrow-derived mesenchymal stem cells (BMSCs) are derived from the mesoderm and within bone marrow [1]. BMSCs have strong abilities to self-renew and can differentiate into various cell types, such as myocytes, adipocytes, chondrocytes, tendon cells, and osteoblasts. A lot of studies have reported that many kinds of traditional Chinese medicine (TCM) were identified as the active compound that could promote the proliferation and differentiation of BMSCs. For example, Chen et al. [2] found extracts from Buzhong Yiqi Decoction (BYD), a well-known ancient tonic prescription in TCM, which had a promoting effect on BMSCs. In their study, BMSCs were isolated and cultured from Sprague-Dawley (SD) rats and treated with BYD extracts. The cell proliferation of BMSCs was quantified with MTT assay. Results showed that BYD extracts had positive effects on the proliferation of rat BMSCs. In addition, many active ingredients within TCM, such as emodin in Polygonum Multiflorum [3] and naringin in Rhizoma Drynariae [4], were demonstrated playing critical roles in promoting the proliferation of BMSCs in vitro.

Osteoarthritis is one of the most common progressive diseases, which affects at least one-third of adults over the age of 55 years [5]. Currently the primary treatment modalities for osteoarthritis are analgesics and nonsteroidal anti-inflammatory drugs, mainly alleviating the symptoms but causing a lot of side effects. BMSCs are an attractive source for tissue engineering and regeneration because of their ability to differentiate in various directions. Numerous studies have reported that BMSCs could form cartilage in vivo and in vitro [6]. And the conditions required for the chondrogenic differentiation of BMSCs in vitro and in vivo are similar, including the involvement of growth factors as well as cell to cell and cell to matrix interactions [7]. Transforming growth factor-*β* (TGF-*β*) family members are potent stimulators of bone formation and have been shown to be positioned in the bone repair site as well as embryonic bone and cartilage formation site [8]. Recent studies demonstrated that TGF-*β* family members played important roles in promoting the expression of cartilage-specific gene [9–11].

According to the theories of TCM, the “Kidney” controls the bone metabolism and generates the marrow. Therefore, the use of “Kidney-tonifying” TCM herbs can contribute to bone health. He at al. [12] reported that Achyranthes bidentata, a traditional Chinese herb commonly used in the treatment of osteoporosis and bone nonunion, could induce the proliferation and osteogenic differentiation of rat BMSCs. Another study revealed that* Atractylodis macrocephalae*, a tonic traditional Chinese herb, could enhance chondrogenic differentiation in BMSCs and the mechanism was dependent on the Sonic Hedgehog (Shh) pathway [13]. A latest study reported that the kidney-reinforcing and marrow-beneficial traditional Chinese medicine- (TCM-) intervened (KRMBTI)-serum had a promoting effect on the proliferation and osteogenic differentiation of BMSCs in rats. In addition, KRMBTI-serum was also shown to promote the expression of TGF-*β*1 [14].

Here, we choose four kinds of Chinese herbal medicine, including Radix Astragali, Salvia, Herba Epimedii, and Saussurea Involucrata, to analyze whether they have positive effects on the proliferation of BMSCs as well as TGF-*β*1-induced chondrogenic differentiation of BMSCs. All the four TCM herbs were often applied to treat osteoarthritis, articular cartilage injury, and osteoporosis with a long history and significant curative effect.

## 2. Materials and Methods

### 2.1. Animals

Sprague-Dawley rats (half male and half female, 4–6 weeks old, weighting 230 ± 20 g) were obtained from the animal center of Nantong University (Jiangsu, China). All rats were housed in the Nantong University animal care facility and were maintained under pathogen-free conditions. Rats were reared in strict accordance with standard operating procedures. Ambient temperature was kept at 22–25°C, humidity of 45% to 55%, light cycle was 12 h light, 12 h darkness. Timely water addition and feeding were managed daily and litter was cleaned once every two days. All experimental procedures were in accordance with the Guidance Suggestions for the Care and Use of Laboratory Animals, formulated by the Ministry of Science and Technology of China. The experimental procedures were approved by the Ethics Committee of the Institute of Wuxi Hospital of Traditional Chinese Medicine.

### 2.2. Preparation of Chinese Herbal Solutions

Radix Astragali, Salvia, Herba Epimedii, and Saussurea Involucrata were provided by the traditional Chinese medicine pharmacy of Wuxi Hospital of TCM (Jiangsu, China). Radix Astragali 90 g, Salvia 45 g, and Herba Epimedii 27 g (these amounts were three-day doses for an adult) were immersed in 500 mL distilled water for 40 minutes, boiled, decocted for 1 hour, and condensed to 60 mL, respectively. Saussurea Involucrata 5.5 g (this amount was a three-day dose for an adult) was cut into pieces and immersed in 500 mL 75% ethanol for 30 days. Then the liquid was filtered out, volatilized at 74°C, and condensed to 60 mL. Crude concentrations of Radix Astragali, Salvia, Herba Epimedii, and Saussurea Involucrata in the Chinese herbal solutions were 1.5 g/mL, 0.75 g/mL, 0.45 g/mL, and 0.09 g/mL, respectively. Chinese herbal solutions were placed in a bottle, sealed, sterilized, and stored at 4°C.

### 2.3. Preparation of Drug-Containing Serums of Chinese Herbal

The Radix Astragali, Salvia, Herba Epimedii, and Saussurea Involucrata groups (5 rats each group) were intragastrically administered with corresponding Chinese herbal solutions (2 mL, twice a day) for 3 consecutive days (based on the clinical oral dose). The control group was intragastrically administered with normal saline (NS). One hour after the final oral administration on the third day, rats were anesthetized by intraperitoneal injection with 3 mg/100 g sodium pentobarbital (Shanghai Reagent Factory, China). Blood was harvested from the heart and centrifuged at 2500 r/min for 25 minutes. The supernatant was harvested, deactivated in a water bath at 56°C for 30 minutes, filtered through a 0.22 *μ*m micro porous membrane, and stored at −20°C.

### 2.4. BMSCs Isolation, Culture, and Chondrogenic Differentiation

4–6-week-old SD rats were sacrificed by cervical dislocation. Both femora and tibias were isolated and rinsed with phosphate-buffered saline (PBS; Gibco, USA). Whole bone marrow cells were collected by repeated washing the bone marrow cavity with low-glucose Dulbecco's modified Eagle's medium (DMEM, Gibco, USA). The flushed mixture was centrifuged, washed, and resuspended in low-glucose DMEM containing 15% fetal bovine serum (FBS; Hyclone, USA) and 1% penicillin-streptomycin (Hyclone, USA). The resuspended cells were seeded into flasks and incubated in a 37°C, 5% CO_2_ environment. The media were changed at 24 hour for the first time and then changed after each 72 hours. The cells were subcultured at a ratio of 1 : 3, and the third generation of cells was prepared for downstream-related experiments. Cell morphology and proliferation were analyzed daily with inverted phase contrast microscopy (LK40, Olympus, Japan).

BMSCs of three passages were used for chondrogenic differentiation. Cells were cultured for 2 weeks in chondrogenic medium, consisting of low-glucose DMEM supplemented with 2 mg/L insulin (Sigma, USA), 3 mg/L transferring (Sigma, USA), 1 mmol/L sodium pyruvate (Institute of Chemical Technology of Nantong University, China), 100 nmol/L dexamethasone (Sigma, USA), and 10 *μ*g/L transforming growth factor *β*1 (TGF-*β*1; PeproTech, USA). Medium was replaced twice a week. Cell morphology and proliferation were analyzed daily with inverted phase contrast microscopy (LK40, Olympus, Japan).

### 2.5. Immunohistochemical Staining for Collagen Type II

Immunocytochemistry was also carried out after 2 weeks of chondrogenic differentiation. For an immunofluorescence stain, TGF-*β*1-induced BMSCs were fixed with 4% paraformaldehyde (Shanghai Reagent Factory, China) for 30 minutes and pretreated with triton-X100 (Sanggo Biotech, China) for 15 minutes. Rabbit polyclonal antibody against collagen type II (1 : 250 dilution; Boster, China) and fluorescein isothiocyanate- (FITC-) conjugated anti-rabbit antibodies (1 : 300 dilution; Boster, China) were used for fluorescent detection of the specific collagen. Fluorescence images were observed and recorded with a fluorescence microscope (YS100; NIKON, Japan). Uninduced BMSCs were used as negative control.

### 2.6. Intervention with Drug-Containing Serums of Chinese Herbal

The third generation of BMSCs were seeded and exposed to culture solution containing 15% FBS plus 1%, 5%, 10% and 15% (v/v) Radix Astragali-, Salvia-, Herba Epimedii-, and Saussurea Involucrata-containing serum, respectively, serving as experimental groups. 15% FBS plus serum of NS group served as control groups. Freshly prepared DMEM containing 15% FBS was used as blank group. Each experimental group and control group were accompanied by a corresponding blank group. The culture media were replaced every 48 hours. TGF-*β*1-induced BMSCs was treated with the same manner above.

### 2.7. Measurement of Cell Survival Rate

On the 7th day after intervention with drug-containing serums of Chinese herbal, the culture fluid was removed. Cells were seeded in 96-well plates at a density of 1 × 10^4^/mL cells per well. And then 20 *μ*L MTT (3-(4,5-dimethylthiazol-2-yl)2,5-diphenyltetrazolium bromide; 5 mg/mL; Sigma, USA) was added to each well and the cells were incubated at 37°C for 4 hours. After that, the supernatant was carefully discarded, and 150 *μ*L dimethyl sulfoxide (DMSO; Sigma, USA) was added to each well with gentle shaking for 10 minutes. After the blue crystals were dissolved in DMSO, the optic density (OD) values were measured with an enzyme linked immunosorbent assay reader (Bio-Rad, USA) at wave length of 492 nm. Five parallel wells were set in each group. An average value of the five wells was calculated and used in following formulas: value of Chinese herbal − enhanced cell proliferation = OD of experimental group − OD of blank group; value of control cell proliferation = OD of control group − OD of blank group.

### 2.8. Statistical Analysis

All experiments were conducted with a sample size of five and values were reported as mean ± standard deviation. The Student's *t*-test was used to analyze the differences between the values of Chinese herbal-enhanced cell proliferation and control cell proliferation in each group. All data were statistically analyzed using SPSS 16.0 software (SPSS, Chicago, IL, USA). A probability value of *P* < 0.05 was considered statistically significant.

## 3. Results

### 3.1. Characterization of Cultured BMSCs and TGF-*β*1-Induced BMSCs Morphology

**Figure 1:**
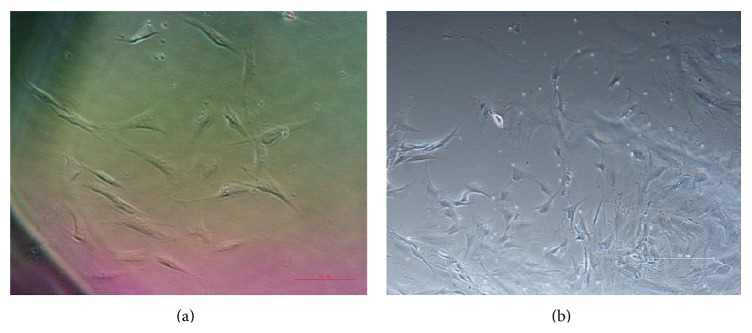
Characterization of cultured BMSCs and TGF-*β*1-induced BMSCs morphology. (a) Normal cultured BMSCs exhibited a spindle-shape (×200); (b) TGF-*β*1-induced BMSCs exhibited larger cell bodies and multiple cytoplasmic projections and appeared a polygonal-shape (×100).

Morphological characteristics of BMSCs in each group were observed daily under an inverted microscope. The newly inoculated BMSCs appeared to be small, round, and mixed with disclike hematocytes. After 24 hours the media was changed for the first time, the majority of cells attached to the surface and appeared in various shapes, including round, rhomboid, spindle, and polygonal shapes. Most of adherent cells exhibited a spindle-shape and colony-type growth after 7 days of culture. Cells grew and exhibited a vortex-like arrangement when covering culture bottle (Figure 1(a)). TGF-*β*1-induced BMSCs exhibited larger cell bodies and multiple cytoplasmic projections and appeared as a polygonal-shape. After 5–8 days the cell bodies increased and the projections extended, exhibiting flattened morphology with the nucleolus split (Figure 1(b)).

### 3.2. Immunofluorescence Analysis

**Figure 2:**
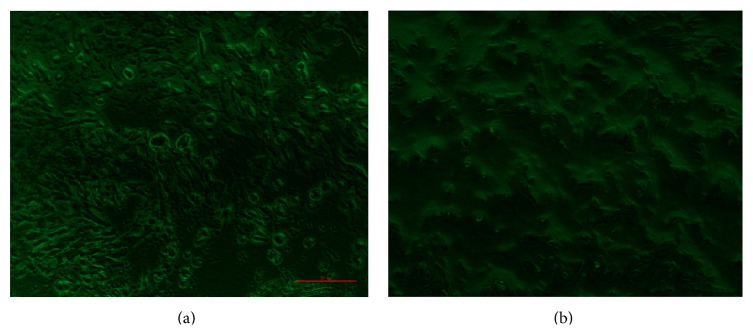
Immunofluorescence staining of Collagen type II protein. (a) Collagen type II protein was strongly detected in the presence of TGF-*β*1 induction (×200); (b) Collagen type II protein was not detectable in the negative control group (×200).

14 days after TGF-*β*1 induction, Collagen type II protein was strongly detected by the immunofluorescence method. Staining of collagen type II protein displayed around the cell membrane (Figure 2(a)). Collagen type II protein was not detectable in the negative control group (Figure 2(b)).

### 3.3. Effects of Drug-Containing Serums of Chinese Herbal on the Cell Proliferation of BMSCs

BMSCs were exposed to culture medium containing 1%, 5%, 10%, and 15% (v/v) concentrations of Radix Astragali-, Salvia-, Herba Epimedii-, and Saussurea Involucrata-containing serum, respectively. OD values of experimental, control, and blank groups were detected with MTT assay after 7 days. The results showed that cell proliferation of BMSCs was significantly enhanced when exposed to culture solution containing 1% and 5% Radix Astragali-containing serum, 1% and 5% Salvia-containing serum, 5% Herba Epimedii-containing serum, and 1%, 5%, and 10% Saussurea Involucrata-containing serum (Table 1).

### 3.4. Effects of Drug-Containing Serums of Chinese Herbal on the Cell Proliferation of TGF-*β*1-Induced BMSCs

TGF-*β*1-induced BMSCs were exposed to culture medium containing 1%, 5%, 10%, and 15% (v/v) concentrations of Radix Astragali-, Salvia-, Herba Epimedii-, and Saussurea Involucrata-containing serum, respectively. OD values of experimental, control, and blank groups were detected with MTT assay after 7 days. The results showed that cell proliferation of TGF-*β*1-induced BMSCs was significantly enhanced when exposed to culture solution containing 1%, 5%, and 15% Radix Astragali-containing serum, 10% and 15% Salvia-containing serum, 5% and 15% Herba Epimedii-containing serum, and 1%, 5%, and 10% Saussurea Involucrata-containing serum (Table 2).

## 4. Discussion

TCM was used to treat a variety of diseases since thousands of years ago. Currently, research on TCM has attracted more and more attention around the world, and there have been many studies which reported traditional Chinese herbs could promote the proliferation of BMSCs [2, 15]. Moreover, it was also reported that Chinese herbal medicine could play an important role in the differentiation of BMSCs. A recent study demonstrated that many kinds of Chinese herbs, including Salvia, Radix Astragali, Ginsenoside, Notoginseng, Musk, and all, could promote neural-directed differentiation of MSCs into nerve cells [16]. Our study demonstrated that Radix Astragali, Salvia, Herba Epimedii, and Saussurea Involucrata could promote the proliferation of BMSCs and TGF-*β*1-induced chondrogenic differentiation of BMSCs in vitro.

Results of our study showed that, in the four different concentrations (1%, 5%, 10%, and 15%) of Chinese herbal-containing serum, 1% and 5% Radix Astragali-containing serum, 1% and 5% Salvia-containing serum, 5% Herba Epimedii-containing serum, and 1%, 5%,and 10% Saussurea Involucrata-containing serum could significantly enhance the proliferation of BMSCs. There was no dose-dependent manner between the proliferation of BMSCs and Chinese herbal drug-containing serum concentrations in our study. Moreover, with respect to the higher concentration of 15%, a lower concentration of 1% or 5% of Chinese herbal drug-containing serum could be more effective in increasing BMSCs proliferation, especially a concentration of 5% in our study.

Actually, our result was consistent with some previous findings that the influence of Chinese medicine on the proliferation of BMSCs was indeed not dependent on the concentrations. A previous study investigated the proliferative effect of Astragaloside IV (AS-IV) in MSCs. AS-IV was one of the major and active components of the Radix Astragali. They are found in the concentration range of 10 to 200 mM AS-IV; the proliferation rates demonstrated optimal effect at 50 mM, much higher than the 200 mM [17]. Another study also reported that there was no dose-dependent manner between MSCs proliferation and AS-IV concentration [18]. In addition, they reported that MSCs proliferation and stem cell factor (SCF) expression were promoted significantly in MSCs stimulated with AS-IV compared with that in MSCs without AS-IV stimulation. SCF was an important hematopoietic growth factor for the growth and proliferation of stem cells. It was also reported that AS-IV could promote the migration of BMSCs by upregulation of CXCR4, which was expressed in early passage of MSCs and served as a vital component in the pathway for stem cell homing [19].

Salvia was a well-known Chinese herb used in the treatment of diseases including osteoporosis. The precise mechanism of Salvia in treating osteoporosis was unclear. Tanshinone IIA was one of the major active phytochemicals isolated from Salvia. It has been reported that tanshinone IIA could inhibit osteoclast differentiation and suppresses bone resorption through disruption of the actin ring, and the precise inhibitory mechanism of Tanshinone VI on osteoclast differentiating might be through inhibiting RANKL expression and NF*κ*B induction [20]. Kwak et al. [21] reported that Tanshinone IIA inhibited PGE2 synthesis in osteoblasts in response to LPS and in turn suppressed osteoclast formation and bone erosion. Furthermore, they found tanshinone IIA suppressed bone loss in mouse models of bone erosion. Our study found that Salvia could enhance the proliferation of BMSCs and promote TGF-*β*1-induced chondrogenic differentiation of BMSCs. Perhaps it could provide a new research idea for Salvia in the treatment of bone diseases. Actually, a Korean study has reported that tanshinone IIA could enhance the commitment of C2C12 cells into osteoblasts and their differentiation through synergistic cross talk between tanshinone IIA-induced p38 activation and BMP-2-induced Smad activation. They hypothesized that the osteogenic activity of tanshinone IIA might lead to the development of therapeutics that benefit bone regeneration and fracture healing [22].

Herba Epimedii is a commonly used traditional Chinese herb for “strengthening the kidney” and providing nutrition to bone. The result of our present study confirmed that Herba Epimedii could increase the proliferation of BMSCs and improve the chondrogenic differentiation of BMSCs in an appropriate circumstance. However, the mechanism was not clear. Interestingly, previous studies have demonstrated that Herba Epimedii could improve osteogenic differentiation in BMSCs [23, 24]. Icariin was considered to be the major pharmacologically active component of Herba Epimedii. Zhai et al. [25] reported that Icariin could enhance the osteogenic differentiation of BMSCs and inhibit the osteoclast formation. And the osteogenic effect of icariin involved the PI3K-AKT-eNOS-NO-cGMP-PKG signal pathway. In addition, a study investigated the effect of Icariin on the genome expression of BMSCs by using stem cell microarray [26]. They found Icariin could regulate the expression levels of 11 genes in BMSCs, including* Gjb2*,* Adam17*,* Adar*,* Alp1*,* Bmp10*,* Bmpr1a*,* Btrc*,* Cdh1*,* Cdkn2a*,* Fgf21*,* Fgfr2*.

Saussurea Involucrata is a rare and precious Chinese herb, most of which grow in 4000 meters above sea level in Tianshan mountain in China. As a traditional Chinese herbal medicine, Saussurea Involucrata has been used in the treatment of rheumatoid arthritis, impotence, irregular menses, and so forth [27]. The finding in our study that Saussurea Involucrata had positive effects on the proliferation and differentiation of BMSCs was described for the first time. Before that, there were nearly no relevant reports. We found 1%, 5%, and 10% Saussurea Involucrata-containing serum could significantly enhance the proliferation of cultured BMSCs as well as TGF-*β*1-induced BMSCs. Perhaps this finding could expand the application of Saussurea Involucrata in terms of bone regeneration and fracture healing. Of course, this requires more in-depth study of the molecular mechanisms.

## 5. Conclusions

In conclusion, our study revealed that Radix Astragali, Salvia, Herba Epimedii, and Saussurea Involucrata could enhance the proliferation of BMSCs and TGF-*β*1-induced BMSCs in vitro. Nevertheless, one limitation existed in our study was that the mechanisms had not been explored. It is the content of our future research. TCM has a long history and a notable curative effect. We suggest that analyzing TCM using modern western research methods can provide a direction for future TCM investigation and contribute to the health of people worldwide.

## Figures and Tables

**Table 1:** Effects of drug-containing serum of Chinese herbal on the cell proliferation of BMSCs determined by MTT.

Drug	*n*	OD of experimental group (OD of control group)				OD of blank group	Value of Chinese herbal-enhanced cell proliferation (value of control cell proliferation)			
		1%	5%	10%	15%		1%	5%	10%	15%
Radix Astragali	5	0.360 ± 0.031	0.379 ± 0.116	0.168 ± 0.046	0.175 ± 0.028	0.063 ± 0.003	0.297 ± 0.034^*∗∗*^	0.316 ± 0.119^*∗∗*^	0.105 ± 0.049	0.112 ± 0.031
Salvia	5	0.447 ± 0.036	0.285 ± 0.015	0.273 ± 0.071	0.189 ± 0.068	0.113 ± 0.015	0.334 ± 0.051^*∗∗*^	0.172 ± 0.030^*∗∗*^	0.160 ± 0.086	0.076 ± 0.083
Herba Epimedii	5	0.208 ± 0.026	0.243 ± 0.026	0.246 ± 0.046	0.218 ± 0.043	0.089 ± 0.007	0.119 ± 0.033	0.154 ± 0.033^*∗∗*^	0.157 ± 0.053	0.129 ± 0.050
Saussurea Involucrata	5	0.287 ± 0.149	0.258 ± 0.030	0.329 ± 0.050	0.292 ± 0.072	0.061 ± 0.003	0.226 ± 0.152^*∗*^	0.197 ± 0.033^*∗∗*^	0.268 ± 0.053^*∗*^	0.231 ± 0.075
Control group	5	0.187 ± 0.002	0.223 ± 0.001	0.263 ± 0.080	0.261 ± 0.070	0.145 ± 0.015	0.042 ± 0.013	0.078 ± 0.016	0.118 ± 0.095	0.116 ± 0.085

Value of Chinese herbal-enhanced cell proliferation = OD of experimental group − OD of blank group; value of control cell proliferation = OD of control group − OD of blank group; comparison between value of Chinese herbal-enhanced cell proliferation and value of control cell proliferation; ^*∗*^ *P* < 0.05, ^*∗∗*^ *P* < 0.01.

**Table 2:** Effects of Chinese herbal drug-containing serum on the cell proliferation of TGF-*β*1-induced BMSCs determined by MTT.

Drug	*n*	OD of experimental group (OD of control group)				OD of blank group	Value of Chinese herbal-enhanced cell proliferation (value of control cell proliferation)			
		1%	5%	10%	15%		1%	5%	10%	15%
Radix Astragali	5	0.390 ± 0.020	0.408 ± 0.001	0.366 ± 0.041	0.528 ± 0.049	0.344 ± 0.051	0.046 ± 0.071^*∗∗*^	0.064 ± 0.050^*∗*^	0.022 ± 0.092	0.184 ± 0.100^*∗∗*^
Salvia	5	0.184 ± 0.013	0.194 ± 0.009	0.383 ± 0.011	0.381 ± 0.072	0.134 ± 0.037	0.050 ± 0.05	0.060 ± 0.028	0.249 ± 0.026^*∗∗*^	0.247 ± 0.109^*∗∗*^
Herba Epimedii	5	0.243 ± 0.011	0.342 ± 0.025	0.275 ± 0.055	0.279 ± 0.015	0.230 ± 0.036	0.013 ± 0.047	0.112 ± 0.061^*∗∗*^	0.045 ± 0.091	0.049 ± 0.051^*∗∗*^
Saussurea Involucrata	5	0.280 ± 0.009	0.358 ± 0.003	0.297 ± 0.061	0.232 ± 0.002	0.229 ± 0.037	0.051 ± 0.046^*∗∗*^	0.129 ± 0.034^*∗*^	0.068 ± 0.098^*∗∗*^	0.003 ± 0.035
Control group	5	0.382 ± 0.026	0.380 ± 0.058	0.386 ± 0.006	0.347 ± 0.031	0.346 ± 0.052	0.036 ± 0.036	0.034 ± 0.11	0.040 ± 0.058	0.001 ± 0.083

Value of Chinese herbal-enhanced cell proliferation = OD of experimental group − OD of blank group; value of control cell proliferation = OD of control group − OD of blank group; comparison between value of Chinese herbal-enhanced cell proliferation and value of control cell proliferation; ^*∗*^ *P* < 0.05, ^*∗∗*^ *P* < 0.01.
